# Supplementary material for: Development of a computer-aided design software for the quantitative evaluation of aesthetic damage
Source: PLoS One. 2019 Dec 18;14(12):e0226322. doi: 10.1371/journal.pone.0226322 (PMC6919621; doi:10.1371/journal.pone.0226322)
Supplement: S1 Code site — 1. (ZIP) [file pone.0226322.s001.zip › [EESC_jr][Projeto]Relatorio_07_21/reports/templates_zip/gentelella-master/vendors/google-code-prettify/README-zh-Hans.html]

Javascript code prettifier


# Javascript code prettifier

# Javascript 代码美容师

## 安装使用

1. 下载 文件- 在你的文件中包含这儿的脚本和样式表(你要保证这儿的 css 和 js 文件按在你的服务器上, 并且调整在 script 和 link标签中的路径)

     ```
     <link href="prettify.css" type="text/css" rel="stylesheet" />
     <script type="text/javascript" src="prettify.js"></script>
     ```

     - 添加`onload="prettyPrint()"` 到你的文件的 body 标签中.- 修改样式表,使用你自己喜欢的颜色.

## 使用方法

在
<pre class="prettyprint">...</pre> 或 <code class="prettyprint">...</code>
中间放上代码片段,它就会自动被美化了.

|  |  |  |  |
| --- | --- | --- | --- |
| The original Prettier|  |  | | --- | --- | | ``` class Voila { public:   // Voila   static const string VOILA = "Voila";    // will not interfere with embedded tags. } ```   ``` class Voila { public:   // Voila   static const string VOILA = "Voila";    // will not interfere with embedded tags. } ``` | | | |

## 常见问题

### 它是为什么语言工作的?

prettify.js中的注释是权威的,但是它的语法分析程序可以在很多语言中使用,包括 C ,
Java, Python, Bash, SQL, HTML, XML, CSS, Javascript, 和 Makefiles.
它在 Ruby, PHP, VB, 和 Awk 中还算可以,而且也可以在 Perl 和 Ruby的合适子集中起作用,但是, 因为注释的约定,它对
Smalltalk, 或 CAML类似的语言起作用.

LISP系列的语言可以使用一个拓展`lang-lisp.js`.

对于 `CSS`,
`Haskell`,
`Lua`,
`OCAML, SML, F#`,
`Visual Basic`,
`SQL`,
`Protocol Buffers`, 和
`WikiText`..也是类似的

如果你想给你喜欢的语言写个拓展版本, 请参阅src/lang-lisp.js ,并写一个包括你的语言的拓展的 发布 和一个测试用例.

### 如何指定我的代码在哪种语言里?

你不需要指定语言环境,因为 `prettyprint()`
会对此进行猜测. 你可以使用 `prettyprint` 这个类通过指定语言的拓展名来指定语言,就像这样:

```
<pre class="prettyprint lang-html">
  The lang-* class specifies the language file extensions.
  File extensions supported by default include
    "bsh", "c", "cc", "cpp", "cs", "csh", "cyc", "cv", "htm", "html",
    "java", "js", "m", "mxml", "perl", "pl", "pm", "py", "rb", "sh",
    "xhtml", "xml", "xsl".
</pre>
```

### 它在混淆代码例子上不起作用吗?

是的. 美化混淆代码就像给小猪涂口红,也就是不在这个工具的范围内.

### 它可以在那些浏览器上工作?

这个工具已经在 IE 6, Firefox 1.5 & 2, 和 Safari 2.0.4 上测试通过. 打开 测试页面 ,看看它能不能在你的浏览器上起作用.

### 有什么改变?

查看 变化日志

### 为什么Prettyprinting 对WordPress中的字符串没用?

很显然,wordpress 在 "smart quoting" 时会改变关闭符号.
这使得关闭符号跟开始符号不配套.

这和复制粘贴代码一样,破坏了美化作用. 去
WordPress's help center 查看更多关于如何关闭插入代码段时的"smart quoting"的信息.

### 如何在我的代码中加入行号? (Out of date -- see English version)

你可以使用 `nocode` 类来标记 span 标记不是代码.

```
<pre class=prettyprint>
<span class="nocode">1:</span> /* This is line 1 of my code
<span class="nocode">2:</span>  * and here's line 2 */
<span class="nocode">3:</span> print("I'm line number 3");
</pre>
```

得到

```
1: /* This is line 1 of my code
2:  * and here's line 2 */
3: print("I'm line number 3");
```

查看一个更完整的例子： issue22
testcase.

### 我得到了这样一条错误信息 "a is not a function" 或 "opt\_whenDone is not a function"

如果你通过事件句柄条用 `prettyPrint` , 把它隐藏到一个函数中.

不要这么写

> `addEventListener('load', prettyPrint, false);`

而要这么写
> `addEventListener('load', function (event) { prettyPrint() }, false);`

这样的话,浏览器就不会把时间对象传递给 `prettyPrint` ,事件对象会让它困惑的.
  
  
  

Last modified: Wed Jan 7 13:25:42 PST 2009
